# A novel approach to low-temperature synthesis of cubic HfO_2_ nanostructures and their cytotoxicity

**DOI:** 10.1038/s41598-017-07753-0

**Published:** 2017-08-24

**Authors:** Neeraj Kumar, Blassan Plackal Adimuriyil George, Heidi Abrahamse, Vyom Parashar, Suprakas Sinha Ray, Jane Catherine Ngila

**Affiliations:** 1 Department of Applied Chemistry, University of Johannesburg, Doornfontein, 2028 South Africa; 2 Laser Research Centre, Faculty of Health Sciences, University of Johannesburg, Doornfontein, 2028 South Africa; 3 DST-CSIR National Centre for Nanostructured Materials, Council for Scientific and Industrial Research, Pretoria, 0001 South Africa

## Abstract

The development of a strategy to stabilise the cubic phase of HfO_2_ at lower temperatures is necessary for the emergence of unique properties that are not realised in the thermodynamically stable monoclinic phase. A very high temperature (>2600 °C) is required to produce the cubic phase of HfO_2_, whereas the monoclinic phase is stable at low temperature. Here, a novel rapid synthesis strategy was designed to develop highly crystalline, pure cubic-phase HfO_2_ nanoparticles (size <10 nm) using microwave irradiation. Furthermore, the as-prepared nanoparticles were converted to different morphologies (spherical nanoparticles and nanoplates) without compromising the cubic phase by employing a post-hydrothermal treatment in the presence of surface modifiers. The cytotoxicities and proliferative profiles of the synthesised cubic HfO_2_ nanostructures were investigated over the MCF-7 breast cancer cell line, along with caspase-3/7 activities. The low-temperature phase stabilisation was significantly attributed to surface imperfections (defects and deformations) induced in the crystal lattice by the desirable presence of Na_2_S·xH_2_O and NaOH. Our work provides unprecedented insight into the stabilisation of nanoscale cubic-phase HfO_2_ in ambient environments; the method could be extended to other challenging phases of nanomaterials.

## Introduction

Hafnium oxide or hafnia (HfO_2_), a group IV-b metal oxide, has emerged as a leading technological material because it shows outstanding physicochemical properties. With a high dielectric permittivity (*κ* = 15–27), high energy barriers relative to SiO_2_, and high thermodynamic stability, HfO_2_ has become a favourite material for next-generation high-*κ* gate dielectrics in microelectronics. Consequently, HfO_2_ has been selected over SiO_2_ for use as the gate dielectric in silicon devices of the Intel™ Core™ family and flash memory devices. It has also attracted commercial interest as a potential candidate for use in ferroelectric random-access memory and non-volatile resistive random-access memory devices^1^. HfO_2_ has a high melting point (~2780 °C), excellent mechanical and corrosion resistance, high neutron absorption coefficient, high density (9.6 g/cm^3^), and low thermal conductivity. These properties permit its use as a refractive protective coating for thermocouples in nuclear applications and as a thermal barrier coating in engines and chemical manufacturing equipment to allow operation at high temperatures or under harsh conditions. Protective coatings of HfO_2_ could potentially be employed on spacecraft surfaces to improve oxidation resistance during re-entry into Earth’s atmosphere^2^. Because it is transparent over the ultraviolet (UV) to infrared (IR) spectral range (band gap *E*_g_ = 5.3−5.9 eV) and its chemically inert, HfO_2_ is a promising host material for optical applications^3^.

Furthermore, HfO_2_ is becoming a favourable material for biological (biosensing, biotransportation, and radiosensitisation)^4–6^, catalytic^7^, fuel cell^8^ (high-temperature electrolytes and fillers), sensing^9^, scintillating^10^, and laser mirror^11^ applications. At present, nanoparticle-based drug discovery and delivery have attracted much attention from researchers and manufacturers. Cancer is one of the leading causes of death globally, with a mortality rate that increases yearly. High-*Z* HfO_2_ nanoparticles show immense potential for future oncology applications. Nanobiotix, a spin-off from the State University of New York and now based in Paris, developed the NBTXR3 product based on HfO_2_ nanoparticles with special coating to permit intracellular high-energy deposition^12^. On exposure to ionizing radiation, the HfO_2_ contained in the NBTXR3 generates large quantities of electrons, which amplify the dose of energy delivered to the tumor^6^. Field *et al*. demonstrated the acute cytotoxicity of HfO_2_ nanoparticles towards HaCat skin cells^13^. Jayaraman *et al*. reported the relatively non-toxic behaviour of differently sized HfO_2_ nanoparticles towards normal 3T3 fibroblast cell lines^14^. However, the cytotoxicity of HfO_2_ nanostructures towards breast cancer cells remains unexplored. Therefore, this study investigated the effects of uncapped and capped HfO_2_ nanoparticles on breast cancer cells. The as-prepared HfO_2_ nanoparticles were modified using 5-fluorouracil (FU), a well-established cancer therapeutic agent, and polyethylene glycol (PEG) to enhance the bioavailability to the cells. The cytotoxicity profile of anticancer drug-conjugated HfO_2_ nanoparticles is of great medical interest.

HfO_2_ adopts many crystallographic structures depending on the temperature. Pure HfO_2_ exists in a monoclinic phase (m-HfO_2_) in standard conditions, transforms to a tetragonal phase (t-HfO_2_) under atmospheric pressure and high temperature (T ≥ 1700 °C), and becomes cubic-phase (c-HfO_2_) with further heating (beyond 2600 °C). The formation of pure t-HfO_2_ and c-HfO_2_ is considered impossible at room temperature (or low temperature), even with ultrafast quenching, because of the comparably low free surface energies^15^. m-HfO_2_ is the most stable phase at low temperature. Furthermore, volume expansion induces large stresses during the phase transformations from cubic to tetragonal and finally to monoclinic, which eventually causes the cracking of HfO_2_ upon cooling from high temperatures. Importantly, quantum-chemical simulation has predicted that the high-temperature phases exhibit superior *κ* (c-HfO_2_: *κ* ~ 27; t-HfO_2_: *κ* ~ 70) compared to the low-temperature phase (m-HfO_2_: *κ* ~ 16) because they possess higher symmetry. The high melting point of HfO_2_ strongly restricts the ability to fabricate it in bulk from conventionally available synthesis methods^1, 15, 16^. Very few reports on the stabilisation of c-HfO_2_ are available in the literature. Researchers have previously stabilised c-HfO_2_ either by incorporating divalent/trivalent impurities into the crystal lattice to create anionic defect centres or by adding metal/metal oxide particles in a compressive shell^16–21^. These obtained crystals were then calcined at high temperatures (650−1500 °C) in a reducing or oxygen gas atmosphere to obtain crystalline HfO_2_^11, 22^. Thin films of cubic/tetragonal phases have also been stabilised by incorporating cations using physical techniques such as atomic layer deposition and ion beam-assisted deposition at temperatures of 500–1000 °C^23^. However, the introduction of dopants and impurities to the crystal lattice affects the fundamental characteristics of pristine HfO_2_ crystals such as change in the bond length, binding energy, or local cation distribution around the oxygen atom, each of which causes changes in the physicochemical properties (including the dielectric behaviour). Heating the crystals at very high temperatures causes deformations in shape and morphologies, weight loss, and the possible formation of agglomerated or bulk crystal structures of HfO_2_. Recently, Rauwel *et al*. demonstrated a non-hydrolytic route that produced low-crystallinity impure c-HfO_2_ nanoparticles using benzylamine as a solvent and heating at 300 °C for two days^24^. Quintero-García *et al*. developed a method combining mechanically assisted metathesis and molten salts (LiCl/KCl) for the synthesis of c-HfO_2_ at 380 °C. This method produced c-HfO_2_ nanoparticles with m-HfO_2_ impurities^25^. Thus, it remains imperative to develop a novel facile route to prepare cubic- and tetragonal-phase HfO_2_ nanostructures at low temperatures and to study the unique shapes and size-dependent properties of these nanostructures, which may permit novel applications.

To the best of our knowledge, this is the first report that presenting a novel solvent-free synthesis route for highly pure crystalline c-HfO_2_ nanoparticles (size <10 nm) in just a few minutes, by the reaction of HfCl_4_ with Na_2_S·xH_2_O under microwave heating. The obtained nanoparticles are further hydrothermally treated with different surface modifiers, such as PEG and FU to achieve nanostructured c-HfO_2_ of different morphologies, and to study the cytotoxicity of the nanostructures towards MCF-7 breast cancer cells.

## Results and Discussion

### Structural and morphological evolution of HfO_2_

**Figure 1:**
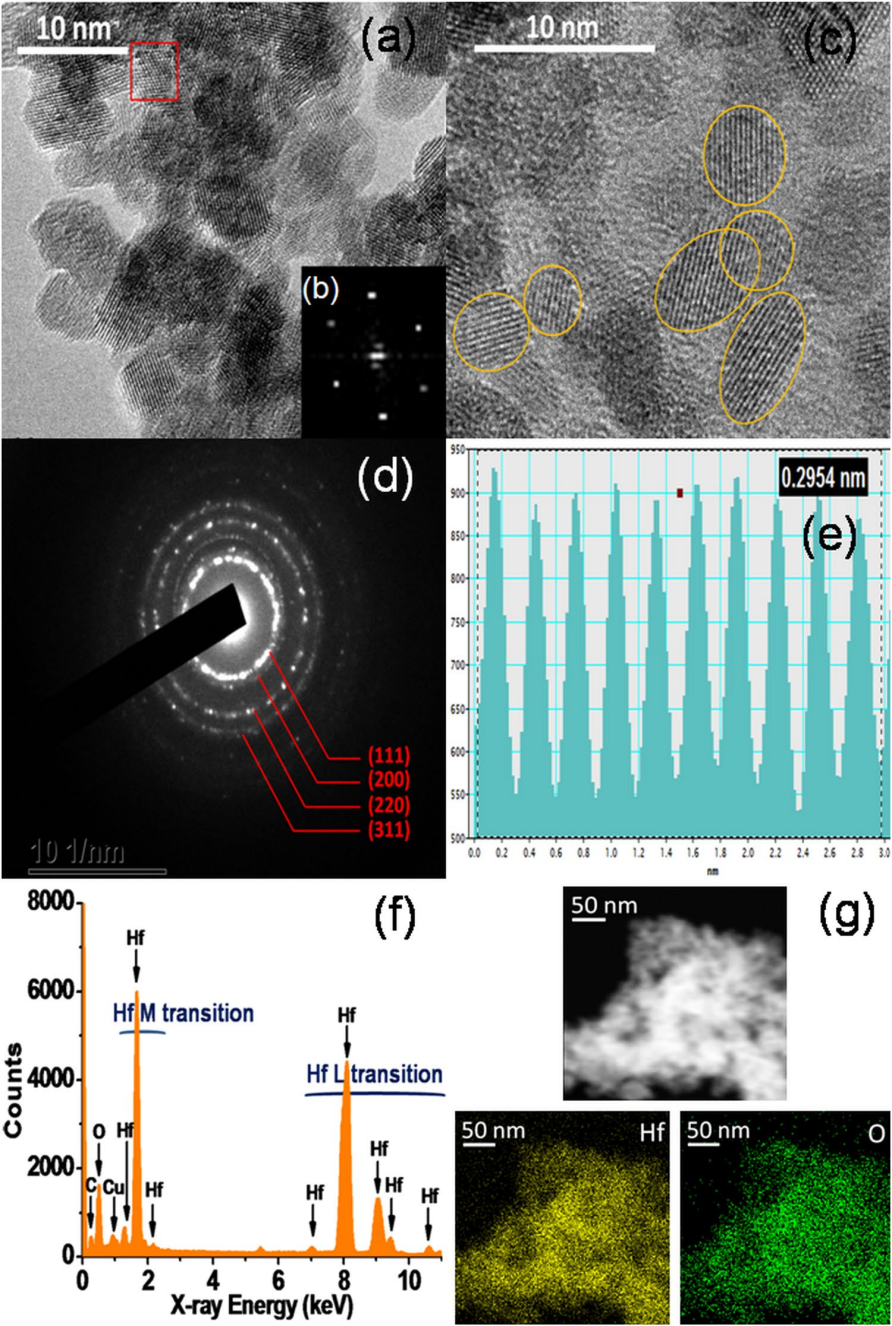
HR-TEM micrographs of the as-prepared c-HfO_2_ nanoparticles (**a**,**c**); (**b**) FFTs show a crystalline ring pattern of the selected area in image (**a**); (**d**) SAED pattern of c-HfO_2_; (**e**) d-spacing calculation using the average of 10 fringes; (**f**) energy-dispersive X-ray (EDX) spectrum; (**g**) scanning tunnelling electron microscopy (STEM) EDX mapping images, exhibiting the homogeneous distribution of hafnium and oxygen.

Fig. 1 presents the morphology, crystallinity, size, and composition of the c-HfO_2_ nanoparticles obtained using the ultrafast microwave synthesis method by reacting HfCl_4_ and Na_2_S·xH_2_O. Transmission electron microscopy (TEM) analysis demonstrates that the present synthesis allows the formation of moderately dispersed nanoparticles with spherical and ellipsoidal shapes (Fig. 1a,c). The formation of clustered and aggregated nanoparticles of HfO_2_ is attributed to the high surface energy and large specific surface area of the particles. A fast Fourier transform (FFT) image shows bright, distinct spot patterns (Fig. 1b) obtained from the imaging of individual isolated nanocrystals from Fig. 1a, characteristic of the crystalline material.

The high-resolution TEM (HRTEM) image (Fig. 1c) indicates explicit lattice fringes that confirm the high crystallinity of the prepared nanoparticles, regardless of the very small dimensions. The HfO_2_ nanoparticles sizes vary from 3.5 to 7 nm. The selected-area electron diffraction (SAED) pattern confirms the polycrystalline nature of the c-HfO_2_, with clear visible spots corresponding to the four main reflection planes of (111), (200), (220), and (311), as expected from the c-HfO_2_ via X-ray diffraction (XRD)^3, 24^.

The d-spacing calculation is performed using the average distances of the ten clearly visible lattice fringes (Fig. 1e﻿). The obtained d-pacing value is 0.295 nm, corresponding to the spacing of {111} planes in c-HfO_2_. STEM was used to perform EDX spectral imaging to explore the chemical composition at nanometer-scale resolution. The obtained EDX spectrum suggests that the HfO_2_ is pure, containing only hafnium and oxygen. The spectrum reveals many hafnium peaks, with lower and higher peaks corresponding to the M and L transitions of hafnium, which overlap with copper peaks. The resulting elemental maps demonstrate the uniform distributions of hafnium and oxygen.

**Figure 2:**
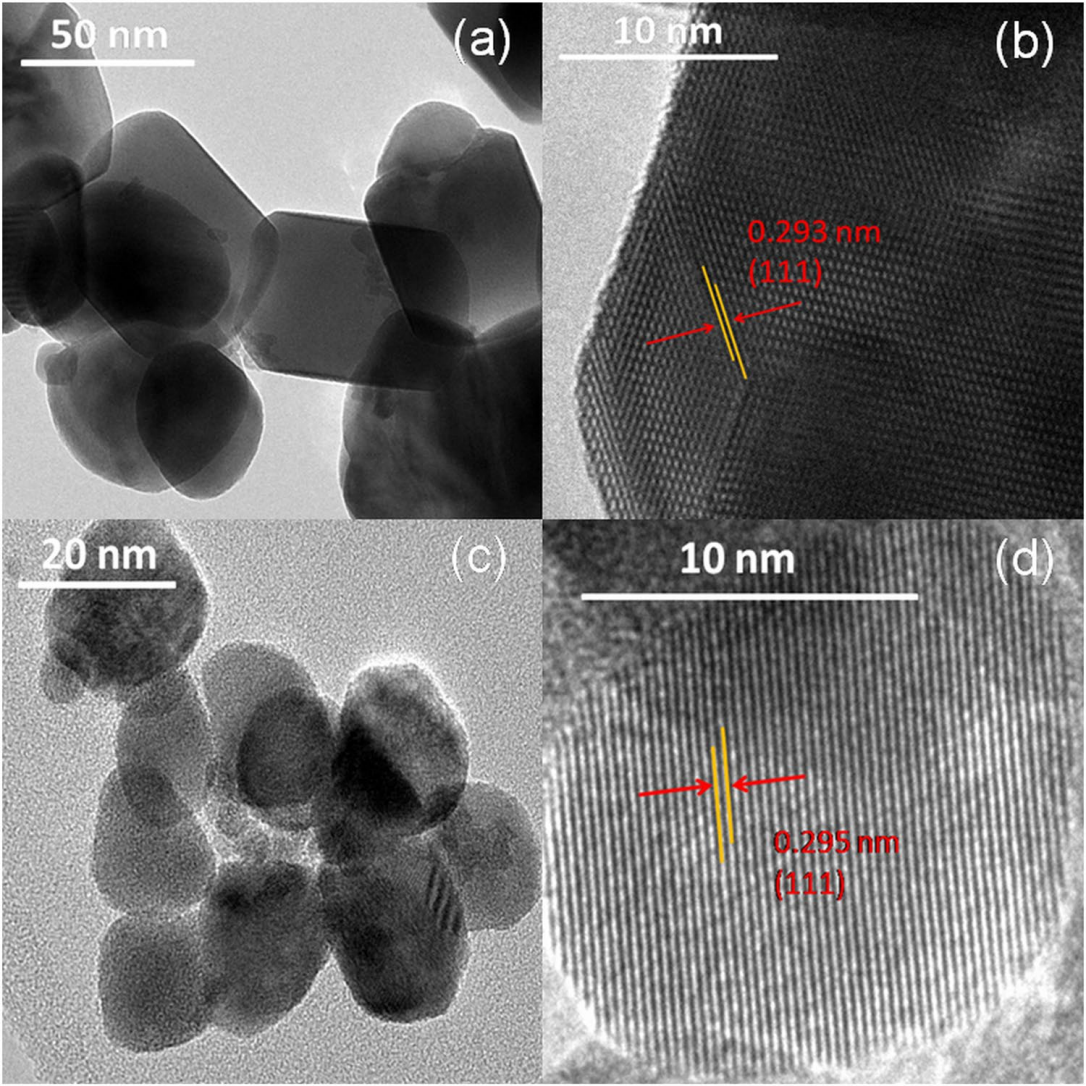
TEM micrographs of c-HfO_2_ nanostructures obtained after treatment in hydrothermal autoclave with PEG (**a**,**b**) and FU (**c**,**d**).

Different morphologies were observed when the freshly dispersed c-HfO_2_ nanoparticles were subjected to hydrothermal treatment using PEG and FU as surface modifiers. Figure 2a exhibits the nanoplates of c-HfO_2_ obtained using PEG. The length of the nanoplates exceeds 50 nm, but the plates have low thickness. c-HfO_2_ nanoparticles grow into larger spherical nanoparticles (16–20 nm) in the presence of FU (Fig. 2c). Distinct lattice fringes with d-spacing of approximately 0.29 nm (the value belonging to the {111} crystal planes of HfO_2_) are obtained in both cases of PEG and FU (Fig. 2b,d).

LaMer *et al*. suggested that the formation of nanocrystals should follow the two stages of nucleation and growth^26^. These two stages are affected by many factors, such as the reactivity of precursors, reaction time, reaction temperature, and ligand types, causing in size variations. The higher reactivity of HfCl_4_ would induce a higher decomposition rate and produce more active units, which in turn would produce more, but smaller, nuclei.

**Figure 3:**
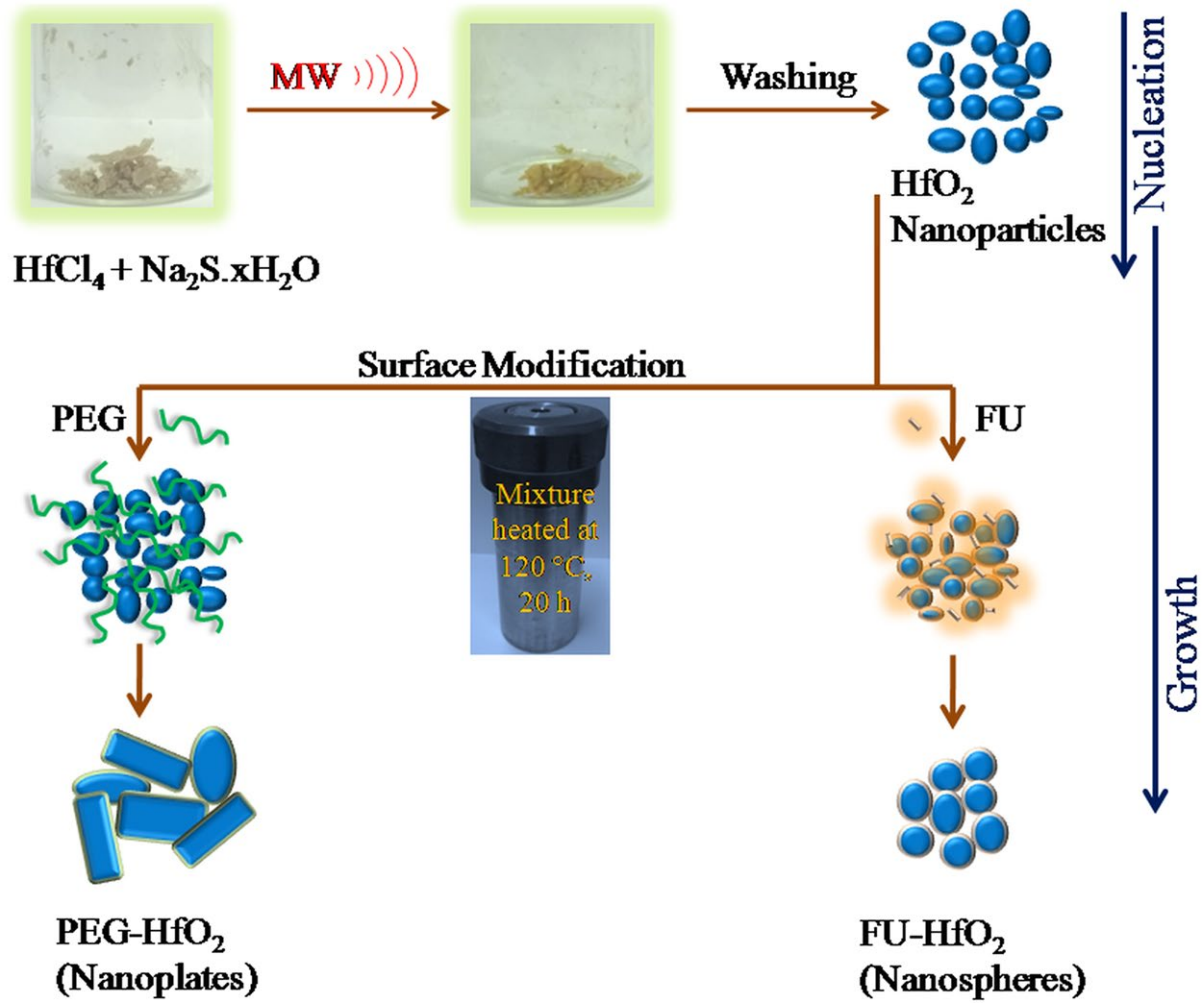
Schematic of the formation of HfO_2_ nanostructures in the presence of PEG and FU (MW represents microwave heating).

Under microwave irradiation, the supersaturation of the reactive units occurs more quickly, which promotes the fast nucleation of HfO_2_ nanoparticles with a high yield. The formed HfO_2_ nuclei spontaneously experience fast growth by diffusion to produce ellipsoidal and spherical nanoparticles^26^. Hydrothermal synthesis allows the selection of reaction parameters, thus permitting phase purity, uniform morphology, size-selective crystal growth, and narrow particle-size distributions^27^. Thus, the growth of the as-prepared c-HfO_2_ nanoparticles occurred under hydrothermal autoclave conditions. The fast reaction kinetics and *in situ* developed high pressures generated different morphologies of c-HfO_2_ in the presence of different ligands. The formed c-HfO_2_ nanoparticles became larger by Ostwald ripening, while PEG was simultaneously selectively adsorbed onto different active crystal facets of HfO_2_ via weak coordination faces, thereby affecting the growing speed and directions of the active faces. With continued feeding of the HfO_2_ particles in hydrothermal conditions, the HfO_2_ nanoparticles partially experienced growth into nanoplate-like nanostructures through oriented aggregation. Furthermore, the PEG-4000 units also formed crown ether-like aggregates via the hydrophobic and hydrophilic molecular regions of PEG and site-specific interactions with water^28, 29^. These aggregates were critical in controlling the growth and direction of the nanoplates through hydrogen bonding^30^. In the presence of FU, large spherical nanoparticles of c-HfO_2_ were formed. Firstly, FU molecules functionalized the c-HfO_2_ nanoparticles via carbonyl and amine moieties. Later, the nanoparticles growth was governed by diffusion and Ostwald ripening of the initial nanoparticles in the carbonyl- and amine-containing environment^31^. One plausible HfO_2_ nanostructures formation mechanism in the presence of different surface modifiers is displayed in Fig. 3.

**Figure 4:**
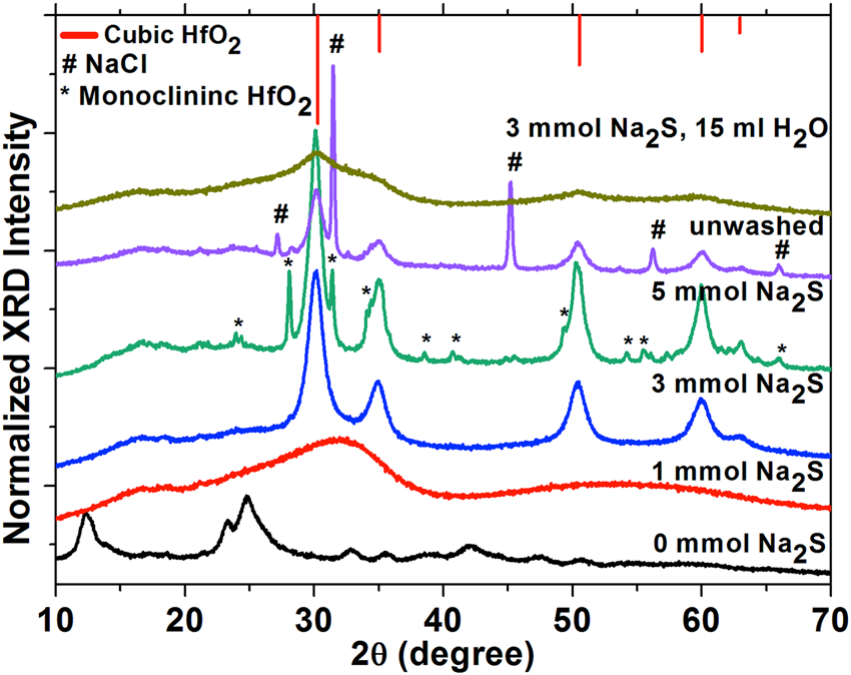
XRD patterns obtained with varied concentrations of Na_2_S·xH_2_O (0, 1, 3, and 5 mmol); without washing after reaction completion (using 3 mmol of Na_2_S·xH_2_O); reaction in aqueous media (used 3 mmol Na_2_S·xH_2_O, and 15 mL water).

The crystallinity and phase characteristics of the HfO_2_ nanoparticles were investigated by XRD patterns, as shown in Fig. 4. The effect of the concentration of Na_2_S·xH_2_O was seen in the crystal structural evolution of HfO_2_. A control reaction was performed to observe the effect of microwave irradiation alone on the HfCl_4_ precursor. HfClO_3_ (JCPDS# 00-032-0422) was formed upon microwave heating of the precursor by the partial oxidation of HfCl_4_ in air (Fig. 4). At 1 mmol of Na_2_S·xH_2_O, the XRD pattern revealed the formation of amorphous HfO_2_. Highly crystalline pure c-HfO_2_ was achieved at 3 mmol Na_2_S·xH_2_O. These HfO_2_ nanoparticles crystallise in a cubic fluorite structure (space group $Fm\bar{3}m$, *a* = 0.509 nm) with reflection planes that are well indexed according to JCPDS# 053–0550. The obtained broadened diffraction pattern suggests very short-range order, originating from crystalline domains with sizes of a few nanometres, and supporting the TEM analysis. The average crystalline size of the c-HfO_2_ nanoparticles is approximately 8–10 nm using the Debye−Scherrer equation, which is consistent with the TEM results. Mixed phases of cubic and monoclinic HfO_2_ (space group *P*2_1_ */c*, *a* = 0.51 nm, *b* = 0.52 nm, *c* = 0.53 nm, *β* = 99.19°, JCPDS# 043–1017) are observed at a high concentration of Na_2_S·xH_2_O (5 mmol)^3, 24^. A reaction was also performed to determine the effect of excess hydroxyl groups (water) on the stabilisation of c-HfO_2_. Amorphous c-HfO_2_ was formed when 15 mL water was introduced to the reaction using 3 mmol of Na_2_S·xH_2_O. Furthermore, it was crucial to study the formation of by-products during the emergence of the c-HfO_2_. An XRD pattern of the unwashed sample shows mixed peaks from the dominant NaCl (JCPDS# 00–088–2300), Na_2_S_2_O_3_, and c-HfO_2_. Thus, the concentration of NaS_2_·xH_2_O is critical for regulating the phase of HfO_2_. The optimized concentration of Na_2_S·xH_2_O (approximately 2–4 mmol per 0.5 mmol HfCl_4_) is necessary to stabilise the cubic phase, possibly by promoting controlled anionic vacancies and hydroxyl unit attachments in the HfO_2_ crystal lattice.

**Figure 5:**
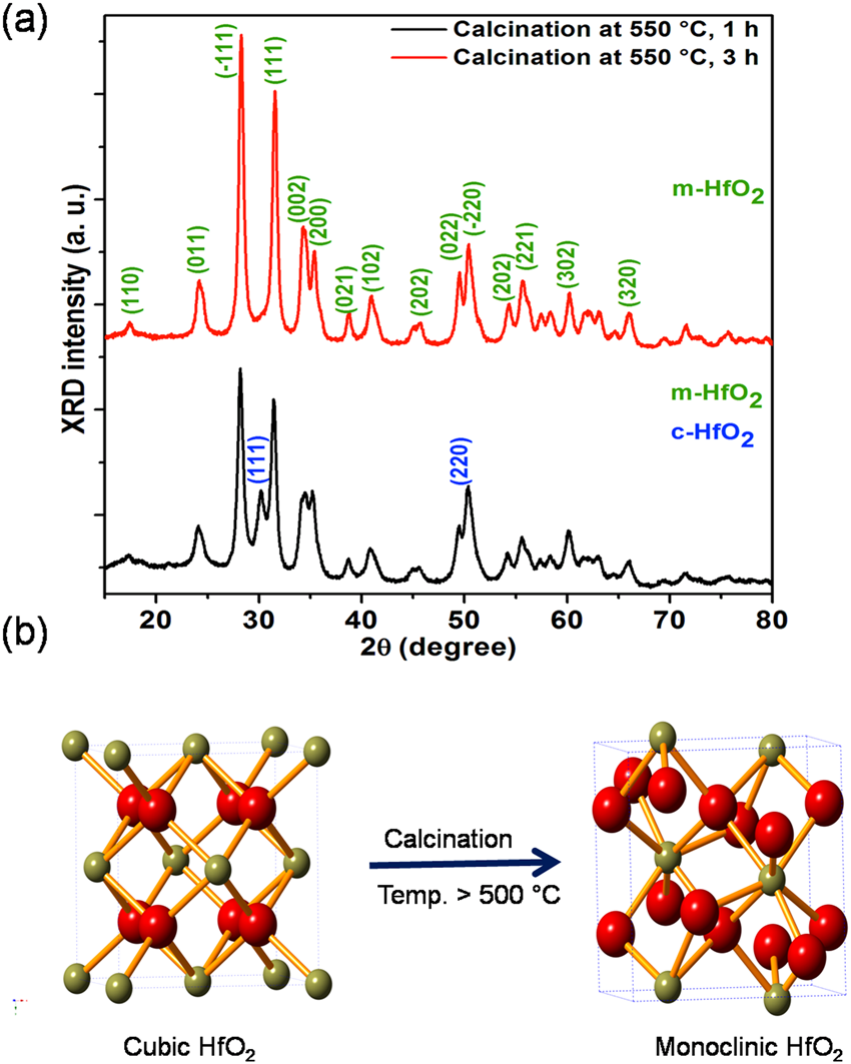
(**a**) XRD patterns show the cubic-to-monoclinic conversion of HfO_2_ with calcination; (**b**) crystallographic structural transformation from cubic-to-monoclinic HfO_2_ upon calcination at temperatures greater than 500 °C (red and olive-green spheres represent O and Hf atoms, respectively).

Furthermore, the as-produced c-HfO_2_ is stable at low temperature (<500 °C) and is converted to m-HfO_2_ upon calcination at temperatures greater than 500 °C for 3 h in air (Fig. 5a,b). Thus, it is noteworthy that the oxygen content is critical for cubic phase stability. The transformation of the lowest-volume phase (cubic) to a higher-volume phase (monoclinic) at high temperatures (>500 °C) has important implications for potential HfO_2_ applications in energy storage systems.

**Figure 6:**
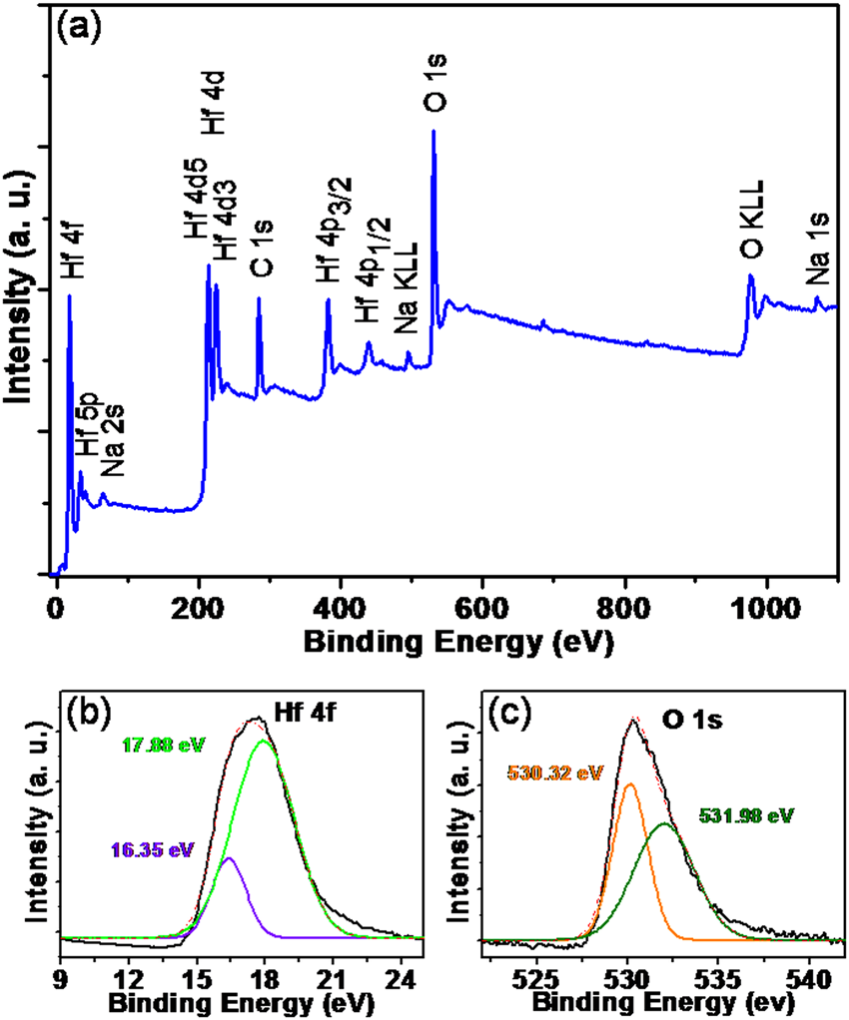
XPS binding energy survey spectrum with marked corresponding peaks (**a**); high-resolution XPS spectra of Hf (**b**) and O (**c**) for synthesised c-HfO_2_.

X-ray photoelectron spectroscopy (XPS) survey spectra and high-resolution spectra of the synthesised c-HfO_2_ were recorded to determine the stoichiometry and chemical states of the elements (Fig. 6). The distinctive peaks of the HfO_2_ bonds at their corresponding binding energies are demonstrated in the XPS survey scan. Peaks at 211.9 and 224.1 eV are assigned to the 4*d*_5/2_ and 4*d*_3/2_ doublet of the Hf 4*d* characteristic spin-orbit doublet of HfO_2_.

The characteristic peak at 380.8 eV is ascribed to the Hf 4*p*_3/2_ photoelectron line of HfO_2_. The appearance of the C 1 *s* peak at 284.4 eV is assigned to adventitious carbon (Fig. 6a). The peaks at 63.5, 496.3, and 1071.7 eV are ascribed to Na 2 *s*, Na KLL, and Na 1 *s*, respectively. The sodium peaks may appear from attached residual impurities of Na_2_S/Na_2_S_2_O_3_ or interstitial/substituted Na^+^ ions. Furthermore, the Hf 4 *f* high-resolution asymmetric spectrum is deconvoluted into two spin-orbit doublet peaks with corresponding 4*f*_7/2_ binding energies at 16.3 and 17.8 eV. In Fig. 6b, the first peak indicates the hafnium suboxides (Hf^x+^), whereas the second peak corresponds to fully oxidised hafnium (Hf^4+^). The O 1 *s* high-resolution spectrum is also deconvoluted into a main Gaussian curve centred at 530.3 eV and a high-energy shoulder peak centred at 531.9 eV (Fig. 6c). The peak at the lower binding energy (530.3 eV) is associated with oxygen deficiencies or vacancies within the HfO_2_ matrix, whereas the higher energy peak can be attributed to surface hydroxides^24^. Thus, the XPS results reaffirm the formation and purity of the c-HfO_2_ nanoparticles. The elemental composition of the HfO_2_ nanoparticles is quantified using XPS, with results shown in Supplementary Table S1.

Based on the above discussion, the proposed plausible reaction for the formation of c-HfO_2_ nanoparticles is as follows:

$$\begin{array}{lll}{{\rm{HfCl}}}_{4}({\rm{s}})+4{{\rm{Na}}}_{2}{\rm{S}}\cdot {{\rm{xH}}}_{2}{\rm{O}}({\rm{s}})+4{{\rm{O}}}_{2}({\rm{g}}) & \underset{2)\,{\rm{Microwave}}\,{\rm{heating}}}{\overset{1)\,{\rm{Mixing}}}{\longrightarrow }} & {{\rm{HfO}}}_{2}({\rm{s}})+4{\rm{NaCl}}({\rm{s}})\\  &  & +2{{\rm{Na}}}_{2}{{\rm{S}}}_{2}{{\rm{O}}}_{3}({\rm{s}})+{{\rm{xH}}}_{2}{\rm{O}}({\rm{g}})\end{array}$$

**Figure 7:**
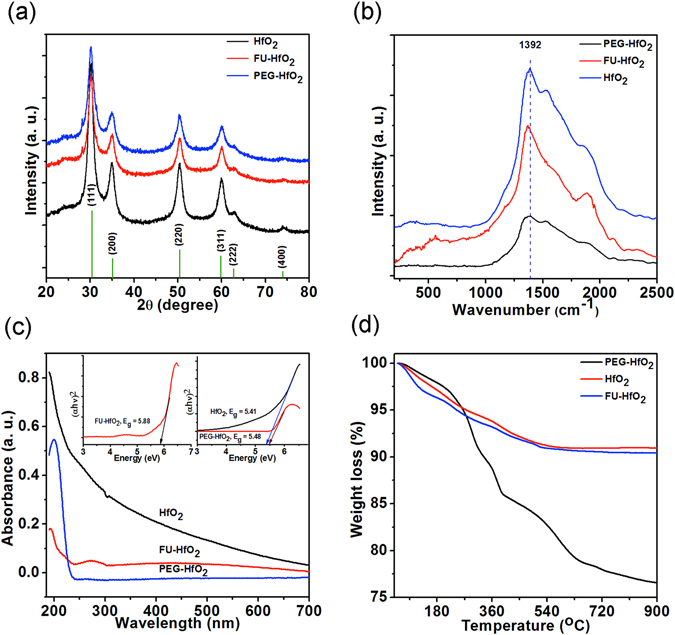
(**a**) XRD patterns, and (**b**) Raman spectra of c-HfO_2_ nanostructures; (**c**) UV-vis absorption spectrum with inset depicting Tauc plots for band gaps; (**d**) thermogravimetric analysis (TGA) curves for c-HfO_2_ nanostructures.

The different morphologies, such as spherical nanoparticles and nanoplates of HfO_2_ are achieved by hydrothermal treatment without compromising the cubic phase of HfO_2_ (Fig. 7a). Spherical nanoparticles are obtained using FU and denoted as FU-HfO_2_, whereas nanoplates are fabricated using PEG and designated as PEG-HfO_2_ throughout the rest of the article. Raman spectra of the c-HfO_2_ nanostructures show broad peaks in the range of 1300–2000 cm^−1^, as shown in Fig. 7b. The observed patterns differ completely from that of m-HfO_2_, possibly because of the breakage of crystal symmetry by point defects or edges in c-HfO_2_^3, 32^.

This behaviour can also be attributed to the high dielectric function of c-HfO_2_. Similar Raman characteristic peaks were also noticed in cubic boron nitride^33^. Furthermore, investigation of the Raman susceptibility of c-HfO_2_ in the IR and UV regions compared to that of m-HfO_2_ as well as the detailed study of the second-order spectra and their correlations with first-principles calculations of the phonon density of states is of interest. Such studies will be conducted systematically in future work.

The UV-vis absorbance spectra of the HfO_2_ nanostructures were recorded from colloidal dispersions in water. For cubic nanoparticles, an abrupt absorption edge accompanied by a very intense absorption in the lower-wavelength region is obtained, as shown in Fig. 7c. The spectra exhibit the exceptional transparency of HfO_2_ throughout the visible and near-IR (NIR) regions. The FU-HfO_2_ sample possesses an extra broad peak at 270 cm^−1^ corresponding to FU, confirming the capping of the HfO_2_ nanostructures^34^. The optical band gaps of HfO_2_, PEG-HfO_2_, and FU-HfO_2_ are calculated as approximately 5.41, 5.48, and 5.88 eV, respectively, using the classical Tauc plot approach (Fig. 7c, insets). The Tauc plot (absorbance coefficient (*αhυ*)^2^ *versus* energy (*hυ*) curve) demonstrates that the absorbance follows the direct allowed transition. An increase in the band gap of HfO_2_ is clearly apparent, possibly from the enhancement of the active role of the many intermediary energy levels or defect states present under the conduction band and the growing crystal sizes of the HfO_2_ nanostructures.

Thermal analysis of the cubic HfO_2_ nanostructures was performed to examine the thermal stability and phase behaviour at high temperatures. Figure 7d exhibits the typical TGA profiles of the HfO_2_ nanostructures. The TGA curves of HfO_2_ and FU-HfO_2_ can be divided into three distinct decomposition stages: The first decomposition at 50–190 °C with a weight loss of 3.45–4.38% can be attributed to water desorption. The weight loss of 6.73–7.05% at 220–365 °C is ascribed to desorption of NaCl, Na_2_S_2_O_3_, and FU (for FU-HfO_2_). At 405–570 °C, a notable weight loss of 9.00–9.32% occurs. This weight loss is ascribed to the cubic-to-monoclinic phase transition of HfO_2_, which is further confirmed by calcination experiments (Fig. 5). For PEG-HFO_2_, three significant weight losses are noticed. A starting weight loss of 2.56% from 50–200 °C is assigned to the water desorption. The second decomposition of 13.83% in the temperature range of 225–392 °C is associated with the desorption of remaining NaCl, Na_2_S_2_O_3_, and PEG^35^. The final major weight loss (21.89%) from 400−650 °C is related to the cubic-to-monoclinic phase transition of HfO_2_.

The Fourier transform IR (FTIR) transmission spectra of HfO_2_ nanostructures (shown in Supplementary Fig. S1) furnish consistent observations. For the HfO_2_ nanoparticles, the broad peak at 754 cm^−1^ is attributed to the Hf−O mode of HfO_2_^36^. The vibrational mode at 950 cm^−1^ is assigned to Hf-OH bonds^37^. The peak at 1631 cm^−1^ belongs to the bending modes of molecular water coordinated to Hf^4+^, whereas the broad band at 3425 cm^−1^ is attributed to O−H stretching in absorbed water molecules^38^. This shows the absence of ligands on the surface of HfO_2_ nanoparticles other than the absorbed water. Some new vibrational modes appear for the nanoparticles capped by PEG and FU. For FU-HfO_2_, absorption bands in the region of 1648–1608 cm^−1^ correspond to C = O and C = C stretching. The peaks at 1469 and 1404 cm^−1^ are the vibrational modes of a multi-substituted pyrimidine ring. The peak at 1103 cm^−1^ can be assigned to the vibration band of C-N^39^. The low-wavenumber peak at 851 cm^−1^ is ascribed to the bending vibration of C-H bonds in the -CF-CH- group^40^. For PEG-HfO_2_, all characteristics bands relating to PEG can be clearly observed, including the O-H stretching at 3368 cm^−1^, the -CH_2_ asymmetric and symmetric stretching of alkyl groups at 2962 and 2930 cm^−1^, respectively, the vibration mode of water associated with PEG at 1598 cm^−1^, the C-H bending mode at 1347 cm^−1^, and the stretching vibration at 1095 cm^−1^ associated with -C-O bonds^29^. The vibration modes relating to Hf-O and Hf-OH bonds are shifted slightly in the FTIR spectra of PEG-HfO_2_ and FU-HfO_2_.

Photoluminescence (PL) spectroscopic studies with λ_ex_ = 320 nm were performed at room temperature to explore invisible agents, such as surface defects or vacancies and surface/trap states in the crystal lattice. Two emission bands are observed in the spectrum at 485 and 524 nm upon excitation at 320 nm (Supplementary Fig. S2). The origin of the blue luminescence at approximately 524 nm is apparently ascribed to defects states such as oxygen vacancies. The emission approximately 485 nm can be assigned to the radiative recombination of charge carriers and band edge defects^24, 41^.

**Figure 8:**
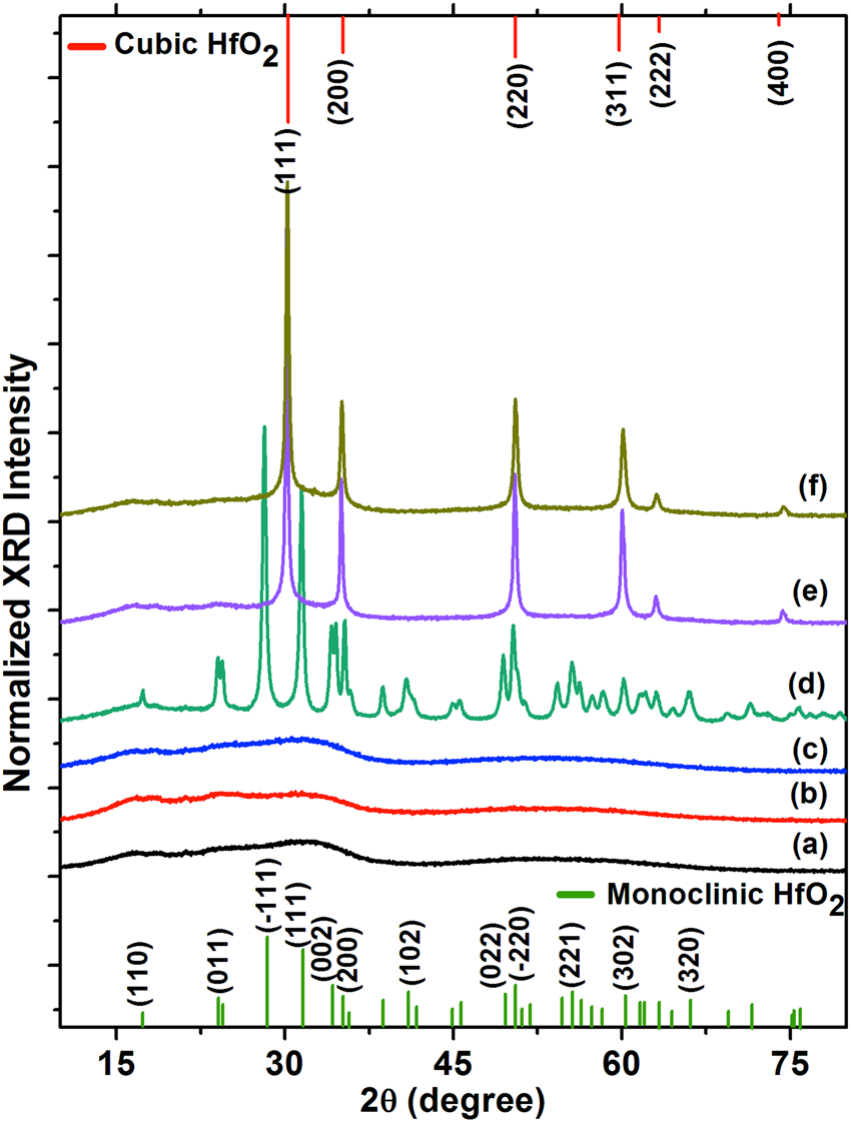
Structural evolution of HfO_2_ when synthesised using varying concentrations of NaOH (4, 6, and 8 mmol). XRD patterns of as-prepared HfO_2_ (**a**–**c**) and after calcining at 550 °C for 1 h (**d**–**e**) with various NaOH concentrations (4, 6, and 8 mmol, respectively).

The effect of NaOH on the structural evolution of HfO_2_ was investigated in detail. Amorphous HfO_2_ was formed in each case with varied concentrations of NaOH of 4, 6, and 8 mmol using the previously developed method (Fig. 8). A different phase of HfO_2_ evolved when the samples were calcined at 550 °C for 2 h. The m-HfO_2_ was achieved at a low concentration of NaOH (4 mmol), whereas the c-HfO_2_ evolved at a high concentration of NaOH. The crystal geometry seemed to depend on the concentration of NaOH, irrespective of the calcination temperature. The main structural effect of the calcination was a sharpening of the diffraction peaks, indicating the enhancement of crystal size. As expected, sample calcination at high temperature had two significant effects: 1) removal of the remaining attached impurities, and 2) lattice configuration (such as crystal growth, coalescence of crystal domains, and rearrangement of lattice defects) and surface restructuring. The above observations imply that a fixed amount of NaOH (5 mmol) is the threshold value for the formation of c-HfO_2_.

Morphological changes were examined to probe the effect of NaOH concentration as well as the influence of temperature using TEM analysis. Almost entirely aggregated nanoparticles were produced by using low and high concentrations of NaOH under normal conditions (Supplementary Fig. S3). m-HfO_2_ nanoparticles, obtained at a low concentration of NaOH, were irregularly interconnected possibly by temperature stresses and diffusion. HRTEM images demonstrated different lattice fringes with *d*-spacings of 0.282, 0.314, and 0.365 nm, corresponding to the {111}, {011}, and {−111} crystal planes, respectively, of m-HfO_2_ (Supplementary Fig. S3c). c-HfO_2_ nanoparticles obtained at a high concentration of NaOH (6 mmol) exhibited mixed morphologies of hollow spheres and clustered/aggregated nanoparticles. Nanocrystals with larger crystalline domains and different continuous orientations were observed in the HRTEM image with the *d*-spacing of approximately 0.295 nm, ascribed to the dominant {111} crystal plane of c-HfO_2_ (shown in Supplementary Fig. S3f). Therefore, crystal growth, coalescence of crystal domains, and restructuring of defects are confirmed by TEM observation of the thermally treated HfO_2_ nanoparticles.

It is considered that the different reducing character of Na_2_S·xH_2_O and NaOH are important in stabilising the c-HfO_2_ at low temperatures. The introduction of anionic vacancies in the crystal system depends strongly on the reducing behaviour of a particular compound. A fixed amount of highly reducing Na_2_S·xH_2_O can generate sufficient anionic defect sites, deformation, and a limited oxygen supply to stabilise c-HfO_2_ at low temperature; however, NaOH fails to stabilise the c-HfO_2_ at low temperature, because it requires a greater oxygen supply with heating. Thus, a sample with a controlled supply of oxygen might favour the smallest-volume structure (c-HfO_2_).

### Cytotoxicity analysis

The cytotoxicity profiles of c-HfO_2_ nanostructures (*viz*. HfO_2_, PEG-HfO_2_, and FU-HFO_2_) were explored using human MCF-7 breast cancer cells. The as-prepared nanostructures were ultrasonicated for 1 h each time before performing the cytotoxicity, proliferation, and cell death analyses.

**Figure 9:**
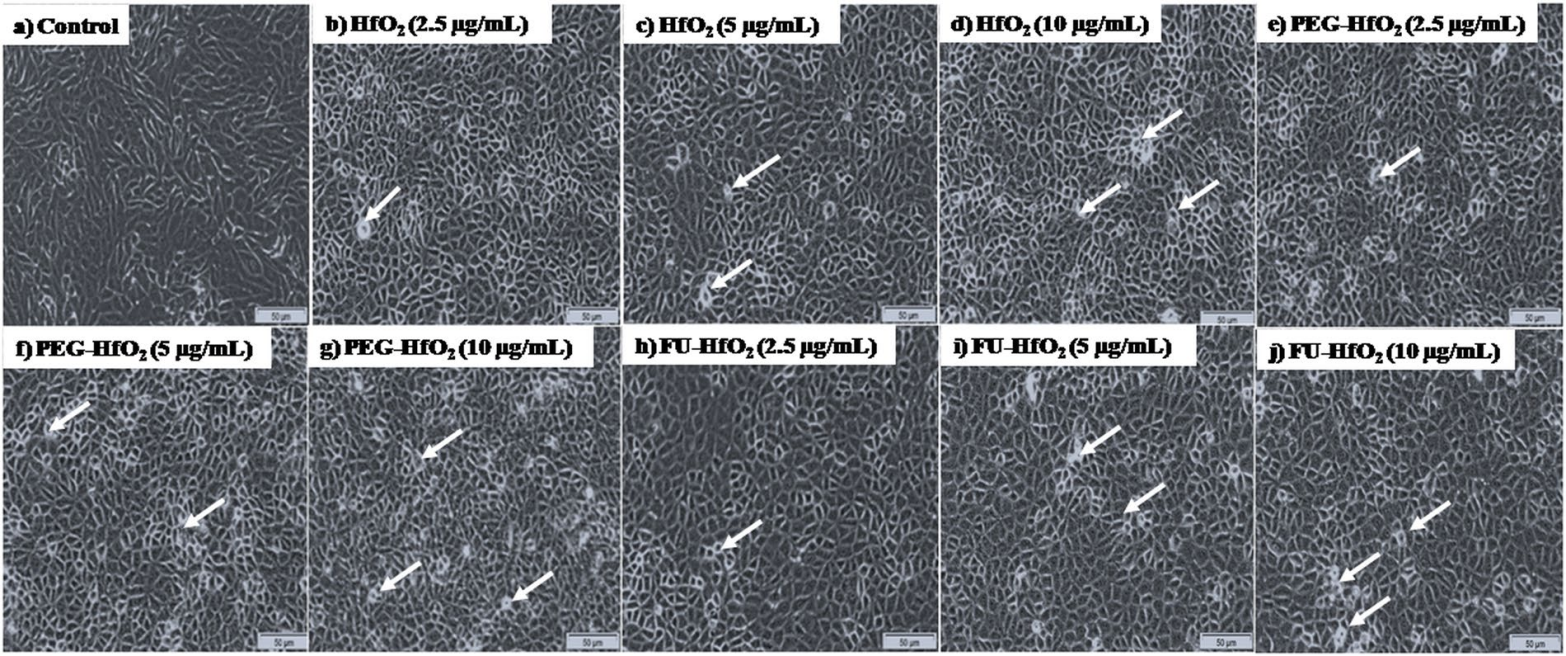
Morphological changes of breast cancer cells (MCF-7) after treatment with HfO_2_, PEG-HfO_2_, and FU-HfO_2_ nanostructures (scale bar for all images is 50 µm; arrows indicate cell death).

Inverted light microscopy displays a considerable changes in the morphology of the MCF-7 cells after the treatment with HfO_2_ nanostructures, as shown in Fig. 9. The morphological variations in the cells after treatment with the HfO_2_ nanostructures support the occurrence of cell death via apoptosis. Compared to untreated cells, the morphologies of the HfO_2_-, PEG-HfO_2_-, and FU-HfO_2_-treated cells after the 24 h incubation period are changed. The numbers of dead cells and amounts of cell debris are increased after the treatment. At higher treatment doses, the numbers of dead cells are increased, and some cells begin to detach from the culture plates and lose shape.

**Figure 10:**
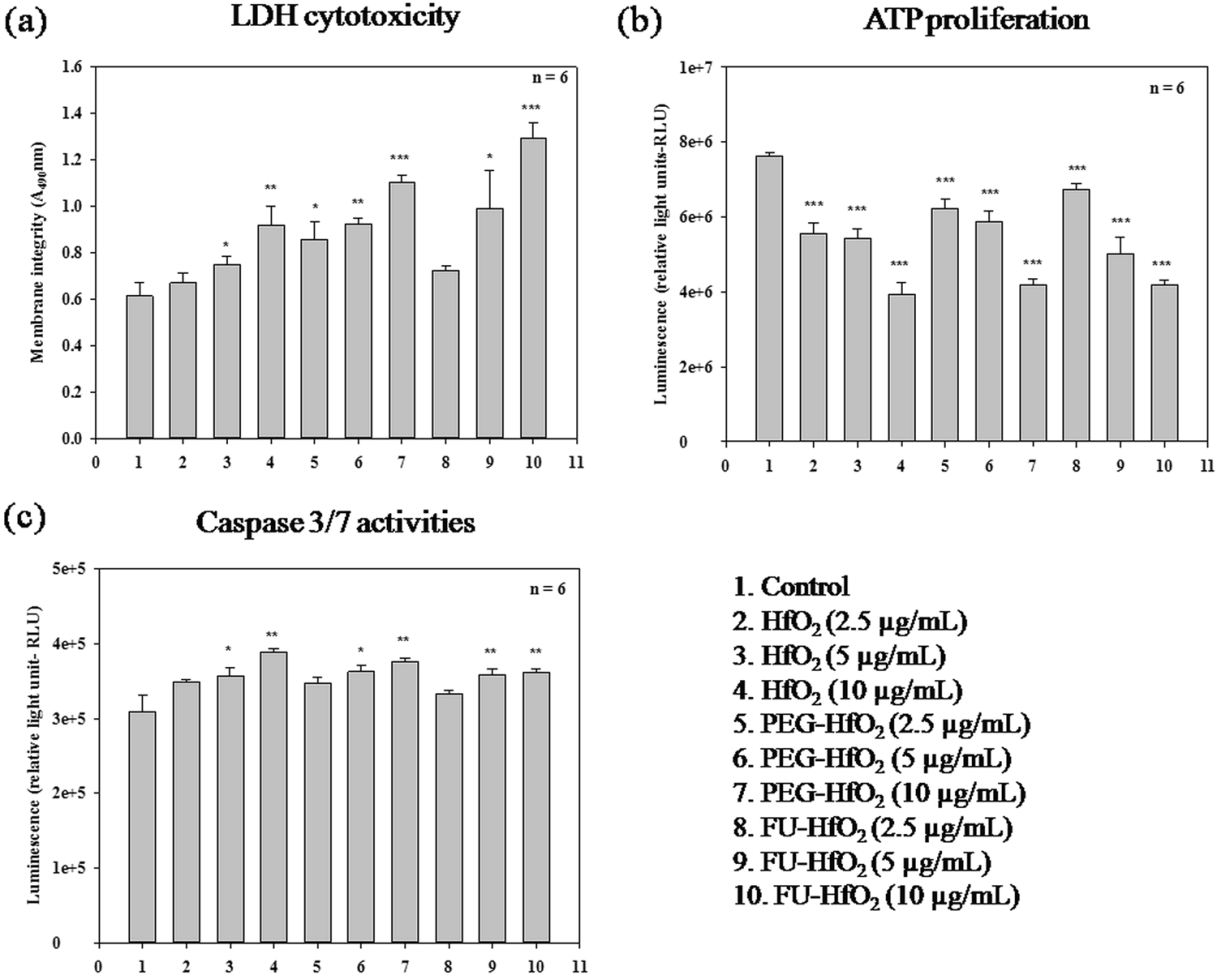
Effect of HfO_2_, PEG-HfO_2_, and FU-HfO_2_ nanostructures on: (**a**) LDH cytotoxicity, (**b**) ATP proliferation, and (**c**) Caspase-3/7 activities. Statistical significance values between the control and treated cells are shown as ^(*)^ *P* < 0.05 ^(**)^ *P* < 0.01 and ^(***)^ *P* < 0.001.

The membrane integrity was assessed by a lactate dehydrogenase (LDH) leakage assay. In this assay, the amount of the cytosolic enzyme LDH released into the culture media was measured as a direct indicator of cytotoxicity. LDH is released upon cell membrane damage, with untreated cells showing reduced LDH release compared to the HfO_2_ nanostructure-treated groups (Fig. 10a). A highly significant (*P* < 0.001) increase in the LDH release is observed in groups treated with 10 µg/mL FU-HfO_2_ and PEG-HfO_2_ with a somewhat significant (*P* < 0.01) increase in those treated with 10 µg/mL HfO_2_ and 5 µg/mL PEG-HfO_2_. The lowest LDH release was observed for lower nanomaterials doses. Better LDH activity directly relates to cell membrane damage, which is further confirmed by the proliferative assay. The intracellular LDH release into the medium is a measure of irreversible cell death due to cell membrane damage^42^.

The changes in morphology, viability, and cytotoxicity of MCF-7 breast cancer cells after 24 h treatment was compared to those of WS1 normal fibroblast cells with and without treatment. The morphology of untreated and treated WS1 cells compared to the MCF-7 cells remains unchanged (Supplementary Fig. S4). The numbers of dead cells in the WS1 cells were smaller; they showed no change in shape and no cellular membrane damage indicating lower toxicity of the compounds towards normal cells compared to that towards MCF-7 breast cancer cells. The HfO_2_ nanostructure-treated WS1 cells remain uniform without loss of membrane integrity; even under higher concentrations, limited numbers of dead cells are apparent. In the trypan blue viability test, 93.25% WS1 cells were viable in the control group; in the treated groups, the viability ranged from 85 to 73.4% (Supplementary Fig. S5a). The viability of MCF-7 cells upon treatment was reduced from 76 to 58% under different concentrations of nanoparticles, compared to 92.75% for untreated cells (Supplementary Fig. S5b). In comparing the LD_50_ values, they were higher in the treated WS1 cells than in MCF-7 cells. The LD_50_ values of HfO_2_, PEG-HfO_2_ and FU-HfO_2_, towards MCF-7 cells were 6.9, 5.6, and 4.2 µg/mL, respectively; in WS1 cells, the LD_50_ values were 10.5, 10.1, and 7.3 µg/mL, respectively. The LDH cytotoxicity results also showed that the HfO_2_ nanostructures were not significantly toxic to normal WS1 cells compared to their behaviour towards MCF-7 cells (Supplementary Fig. S5c).

The toxicity of the surface modifier, *viz*. FU was also tested on MCF-7 cells. Significant morphological changes were observed in the MCF-7 cells after 24 h of treatment with FU, clearly showing losses in cell numbers and membrane damage indicating cell death (Supplementary Fig. S6). The trypan blue viability results also showed decreased viability compared to that of the control cells. In the control group, 87.75% of WS1 cells remained viable; in the treated groups the viability varied from 68.0 to 62.13% (Supplementary Fig. S7). The LD_50_ value of FU towards MCF-7 cells was 5.08 µg/mL, higher than that of the FU-HfO_2_ nanoparticles. This observation shows that the conjugation of the anticancer drug (FU) with HfO_2_ enhanced the toxicity of FU-HfO_2_ towards MCF-7 cells.

The metabolic activity of the cells was determined by measuring the levels of cellular ATP content. ATP, a marker for cell viability and proliferation, is present in all metabolically active cells^43^. The metabolic activity in MCF-7 cells was higher, which was clear from the higher ATP levels in the untreated cells. The HfO_2_-, PEG-HfO_2_-, and FU-HfO_2_-treated cells at all tested doses significantly (*P* < 0.001) decreased the cell growth in a dose-dependent manner (Fig. 10b).

Programmed cell death by apoptosis is generally characterised by distinct morphological characteristics and energy-dependent mechanisms. Caspase-3 and -7 are important executioner cysteine protease enzymes. The measure of caspase-3/7 activity in the cells directly reflects caspase-dependent intrinsic apoptosis^44^. The results of our experiments clearly showed that treatments with the HfO_2_, PEG-HfO_2_, and FU-HfO_2_ nanostructures significantly induced apoptosis in MCF-7 breast cancer cells. There was a significant (*P* < 0.01) increase in caspase-3/7 activities observed in group treated with 10 µg/mL HfO_2_, PEG-HfO_2_, and FU-HfO_2_, as well as those with 5 µg/mL FU-HfO_2_ (Fig. 10c), compared to that in the untreated cells. Lower doses (2.5 µg/mL) of the nanostructures showed little caspase release upon treatment, compared to that with the control cells. In order to identify the pathway of cell death induced in the MCF-7 cells treated with HfO_2_ nanostructures, we investigated the specific activation of two effects or caspases (caspase-3 and -7). The HfO_2_ nanostructures induced the up-regulation of caspase-3 and -7; this demonstrates that nanoparticles of different compositions and sizes can generate different degrees of intracellular responses, which may cause cell death via the induction of apoptosis. In this study, the ATP proliferation results did not exactly correlate with the results of LDH cytotoxicity and caspase activities; however, the significant LDH toxicity and caspase activities observed for cells treated with higher doses of PEG-HfO_2_ and FU-HfO_2_ are striking.

Owing to the strong influence of various parameters (such as surface charge, size, and morphology) and the physicochemical properties of the nanomaterials on cytotoxicity, understanding the exact mechanism of cellular toxicity induced by nanomaterials remains challenging. In the literature, numerous mechanisms of nanomaterial toxicity have been demonstrated, including reactive oxygen species (ROS) generation, cytokine induction, oxidative stress, apoptosis, and DNA damage during *in vitro* studies at the cellular level. In various studies, the cytotoxicities of metal oxides (*viz*. ZnO, TiO_2_, CuO, MgO, and MoO_3_) are attributed to oxidative stresses and the overproduction of ROS^29, 45, 46^. Elsewhere, the photocatalytic properties of TiO_2_ nanoparticles were shown to induce cell death^47, 48^. Thus, nanoparticle-induced cell death, or cytotoxicity, is governed by several factors.

In the present study, the cubic HfO_2_ nanostructures demonstrated significant cytotoxicity towards breast cancer cell lines (MCF-7). The high cytotoxicity of the HfO_2_ nanostructures is achieved by their high metal-ion release capability, size, structural characteristics, active surface availability, defects, and high adsorption tendency. The possible mechanisms for MCF-7 cell death on exposure to HfO_2_ nanostructures are overproduction of ROS and oxidative stress, which ultimately induces apoptosis via losses in mitochondrial membrane potential. This mechanism of oxidative stress is further substantiated by the caspase-3/7 activities. From the above cytotoxicity analysis, the cytotoxicity order in the HfO_2_ nanostructures was observed as follows: FU-HfO_2_ > PEG-HfO_2_ > HfO_2_. The surface-modified HfO_2_ nanostructures (FU-HfO_2_, PEG-HfO_2_) exhibited better cytotoxicity because of enhanced cellular uptake, biocompatibility, stealth properties, defects/vacancies, and physiological environment. The FU-HfO_2_ sample showed excellent cytotoxicity in the LDH assay and also showed higher caspase activities, possibly from the coating of the cancer drug FU, which enhanced its ability to penetrate the cell membrane^49^.

HfO_2_ nanoparticles have been demonstrated as efficient radio-enhancers that could be applicable to most solid tumors^6, 12, 49, 50^. In the future, combination therapy using FU-HfO_2_ nanoparticles along with radiotherapy could reduce side effects and enhance the efficacy of treatment for cancer patients. The best combination could be screened out by combining different available potent anticancer agents with HfO_2_ to achieve better therapeutic attributes.

## Conclusion

A strategy is presented to realise the goal of achieving high-quality cubic HfO_2_ nanoparticles with excellent properties in an ambient environment by employing microwave heating. The concentration of Na_2_S·xH_2_O or NaOH was optimised to stabilise the cubic phase. Na_2_S·xH_2_O demonstrated better facility in stabilising c-HfO_2_ at low temperature. Structural and morphological characterizations by various techniques such as TEM, elemental analysis, XRD, UV-Vis, FTIR, TGA, XPS, Raman spectroscopy, and PL measurements confirmed the formation of the HfO_2_ cubic polymorph. The as-prepared c-HfO_2_ nanoparticles were subjected to post-hydrothermal treatments to achieve different morphologies (nanoplates and spherical nanoparticles) of c-HfO_2_ without compromising the phase composition using surface modifiers of PEG, and FU (a cancer drug), respectively. The obtained nanostructure samples (HfO_2_, PEG-HFO_2_, and FU-HFO_2_) were tested for cytotoxicity and cell death induction towards MCF-7 breast cell lines. FU-HFO_2_ showed excellent cytotoxicity in the LDH assay and also showed higher caspase activities from the coating of FU, which enhanced its ability to penetrate the cell membranes. A significant dose-dependent variable effect on LDH cytotoxicity and ATP proliferation was observed with increasing concentrations of all tested nanostructures. Thus, the coating of FU on c-HfO_2_ could augment the biological response of the cancer cells, consequently enhancing the efficacy of cancer therapies. Furthermore, the HfO_2_ nanostructures were relatively non-toxic to WS1 normal fibroblast cells. These findings offer a new rapid method to stabilise the cubic phase of HfO_2_ at low temperatures; this method could be expanded to realise the high-temperature phase stabilisation of other nanomaterials. This facile scalable synthesis of cubic HfO_2_ may also permit the examination of its new properties and the design of novel applications.

## Methods

### Materials

All the chemicals purchased were of analytical grade and were used as received. HfCl_4_ (98%), Na_2_S·xH_2_O (≥60%), FU (≥99% HPLC), NaOH (≥98%, pellets), and PEG−4000 were procured from Sigma-Aldrich, South Africa.

### Synthesis of HfO_2_ nanostructures

HfO_2_ nanoparticles were synthesised by using a microwave assisted reduction−oxidation method. First, the starting materials (HfCl_4_ (0.5 mmol)) and Na_2_S·xH_2_O (1, 3, and 5 mmol) were hand-mixed thoroughly in a porcelain mortar with a pestle for ~3–4 minutes. A semi-solid grey paste was formed after mixing. In the second step, the obtained semi-solid paste was exposed to microwave irradiation in a domestic microwave oven (using a power of 80% of 800 W) for 4 min at 20 s intervals. The obtained yellow-orange solid product (Supplementary Fig. S8) was thoroughly washed with water by centrifugation to remove by-product impurities, and the final precipitate was dried at 60 °C for 8 h in an air oven. A series of reactions was also performed to investigate the effect of NaOH (2, 4, and 6 mmol) instead of Na_2_S·xH_2_O on the phase, crystallinity, and structure of HfO_2_. Various reaction conditions for the synthesis of cubic HfO_2_ nanostructures are summarised in Supplementary Table S2.

For the surface modification, the precipitate obtained just after centrifugation was added to 30 mL deionized water and was ultrasonicated for 20 min. PEG/FU (0.350 g) was added to the above dispersed HfO_2_ nanoparticles and stirred for 15 min. Then, the as-prepared solution was transferred into a Teflon-lined stainless-steel hydrothermal autoclave and heated at 120 °C for 20 h in an electric oven. The obtained sample was then washed with deionized water and ethanol by centrifugation and dried at 60 °C.

### Materials characterization

Powder X-ray diffraction (XRD) patterns of the as-prepared HfO_2_ nanostructures were recorded using a Philips PANalytical X’Pert X-ray diffractometer at 40 kV and 40 mA with Cu-Kα radiation (0.15418 nm). A HRTEM (JEOL JEM-2100) equipped with a STEM for EDX spectroscopy was employed to study the morphologies, crystallinities, and size distributions of the materials. Micro-Raman spectroscopy (JASCO, NRS-3100) with a 532 nm solid-state primary laser as an excitation source was used to perform the phonon vibrational study at room temperature. XPS measurements were performed on a Kratos AXIS Ultra device with an Al monochromatic X-ray source (1486.6 eV). The PL spectroscopic measurements were taken using a PerkinElmer (LS 45 Fluorescence Spectrometer, 230 V) instrument at room temperature. UV absorbance measurements were performed on a Shimadzu UV-2450. The thermo-stabilities of the materials were evaluated using a Perkin Elmer TGA (4000) at a heating rate of 10 °C/min under a N_2_ atmosphere over a temperature range of 50–900 °C. FTIR measurements were performed using a Perkin Elmer Spectrum 100 FTIR spectrophotometer, adopting the KBr pellet method.

### Cell lines and maintenance

Human skin fibroblast monolayer cultures (WS1-ATCC CRL1502) were grown in a modified Eagle’s minimal essential medium (Invitrogen 32360–026) with 2 mM L-glutamine (Gibco, 25030), 1 mM sodium pyruvate (Gibco, 11360), 0.1 mM nonessential amino acids (Gibco, 11140), 1% amphotericin-B (Gibco, 104813), 1% penicillin–streptomycin (Gibco, 15140) and 10% v/v foetal bovine serum (FBS; Gibco, 306.00301). Once the cells reached 80% confluence, they were harvested and seeded in a 3.5 cm-diameter culture plate overnight at a concentration of 6.5 × 10^5^ cells for experimental purposes. MCF-7 (ATCCHTB-22, Switzerland) human breast cancer cells were grown in Dulbecco’s Modified Eagle Medium (DMEM) culture. The DMEM was supplemented with 10% FBS (Gibco 306.00301, Thermo Fisher Scientific, South Africa), 1% penicillin−streptomycin, and 1 µg/mL amphotericin-B (PAA Laboratories GmbH, Germany, P11–010 and P11–001). When the cells exceeded 80% confluence, they were washed with Hank’s Balanced Salt Solution (Invitrogen, 10–543 F, Thermo Fisher Scientific, South Africa) and detached using 1 mL/25 cm^2^ of TrypLE express reagent (Gibco, 12604, Thermo Fisher Scientific, South Africa). For the experiments, the cells were seeded in a 3.5-cm-diameter culture plate at a final concentration of 5 × 10^5^ cells/plate. The cultures were incubated at 37 °C with 5% CO_2_ and 80% humidity.

### Cellular morphology and cytotoxicity

The influence of the HfO_2_ nanoparticles on the cellular morphology was determined using an inverted microscope (Wirsam, Olympus CKX 41) after 24 h of treatment. A Cyto-Tox96^®^ X assay (Anatech, Promega G 400, Anatech Analytical Technology, South Africa) was used to measure the cytotoxicity by quantifying the free lactate dehydrogenase (LDH). The damaged cells released LDH to the culture media. The membrane integrity was assessed by estimating the amount of LDH present in the culture media. 50 µL of LDH reagent was added to a 50-µL cell culture medium and incubated in the dark at room temperature for 30 min. The colorimetric compound was measured at 490 nm with the VICTOR3™ plate reader (Perkin-Elmer, South Africa).

### Adenosine triphosphate (ATP) luminescent proliferative assay

The rate of cell proliferation was measured by CellTiter-Glo^1^ luminescent assay (Promega, G7571, Anatech Analytical Technology, South Africa) by quantifying the ATP levels of the treated cells. An equal volume (50 µL) of ATP reagent and cell suspension was incubated at room temperature for 10 min in the dark. The luminescent signal produced was measured with the VICTOR3™ plate reader (Perkin-Elmer, South Africa).

### Caspase-3/7 activities

Caspase-3 and -7 are apoptotic protease enzymes involved in the cell execution phase. The activities of caspase-3 and -7 were determined using Caspase-Glo 3/7 luminescent assay (Whitehead Scientific, Bracken fell, South Africa; Promega G8091). The re-suspended cells (50 µL) were added to an equal volume of Caspase-Glo-3 and -7 substrate reagent in a luminescent plate and incubated at room temperature for 3 h. The luminescent signal was measured with the VICTOR3™ plate reader.

### Statistical analysis

The cells between 15 and 20 passages were chosen for the study. To determine the actual significance, the experimental samples were compared with control cells by one-way analysis of variance (ANOVA). The statistical significances between the control and treated cells are shown as ^(*)^ *P* < 0.05 ^(**)^ *P* < 0.01 and ^(***)^ *P* < 0.001.

## Electronic supplementary material

Supplementary Data A novel approach to low-temperature synthesis of cubic HfO2 nanostructures and their cytotoxicity
