# Supplementary material for: A novel approach to low-temperature synthesis of cubic HfO2 nanostructures and their cytotoxicity
Source: Sci Rep. 2017 Aug 24;7:9351. doi: 10.1038/s41598-017-07753-0 (PMC5570928; doi:10.1038/s41598-017-07753-0)
Supplement: Supplementary file 1 — Supplementary Data A novel approach to low-temperature synthesis of cubic HfO2 nanostructures and their cytotoxicity [file 41598_2017_7753_MOESM1_ESM.pdf]

Supplementary Data

A novel approach to low-temperature synthesis of cubic HfO_2 nanostructures and their cytotoxicity

Neeraj Kumar*[1], Blassan Plackal Adimuriyil George[2], Heidi Abrahamse[2], Vyom Parashar[1], Suprakas Sinha Ray[1,3], Jane Catherine Ngila*[1]

[1]*Department of Applied Chemistry, University of Johannesburg, Doornfontein 2028, South Africa,*

[2]*Laser Research Centre, Faculty of Health Sciences, University of Johannesburg, Doornfontein 2028, South Africa*

[3]*DST-CSIR National Centre for Nanostructured Materials, Council for Scientific and Industrial Research, Pretoria 0001, South Africa*

***Corresponding authors:**

E-mail Address: *ynk.neeraj@gmail.com* (Neeraj Kumar)**,** *jcngila@uj.ac.za* (Jane Catherine Ngila)

Contents

Table S1. Elemental composition of HfO_2 nanoparticles obtained from XPS analysis. 3

Figure S1. FTIR transmission spectra of cubic HfO_2 nanostructures. 3

Figure S2. Photoluminescence (PL) spectra of cubic HfO_2 nanostructures recorded at room temperature. 4

Figure S3. TEM micrographs of as-prepared HfO_2 (a) and after calcining at 550 °C (b, c) using NaOH (4 mmol); as-prepared HfO_2 (d) and after calcining at 550 °C (e, f) using a high concentration of NaOH (6 mmol). 4

Figure S4. Morphological changes of WS1 cells after treatment with HfO_2, PEG-HfO_2, and FU-HfO_2 nanostructures; (arrows indicate the cell death). 5

Figure S5. Effect of HfO_2, PEG-HfO_2, and FU-HfO_2 nanostructures on: (a) Trypan blue viability of MCF-7 cells, (b) Trypan blue viability of WS1 cells, and (c) LDH cytotoxicity of WS1 cells. Statistical significance values between the control and treated cells are shown as P <0.001 (***). 6

Figure S6. Morphological changes of MCF-7 cells after treatment with 5-fluorouracil (FU) (scale bar for all images is 50 µm). 7

Figure S7. Effect of 5-fluorouracil (FU) on: (a) Trypan blue viability of MCF-7 cells. Statistical significance values between the control and treated cells are shown as P <0.001 (***). 7

Figure S8. Digital images represent the different synthesis stages of cubic HfO_2 nanostructures. 8

Table S2. Summarised reaction conditions for the synthesis of cubic HfO_2 nanostructures 8

Table S1. Elemental composition of HfO_2 nanoparticles obtained from XPS analysis.

Element	Atomic %
Hafnium	18.5
Oxygen	38.7
Carbon	41.2
Sodium	1.6

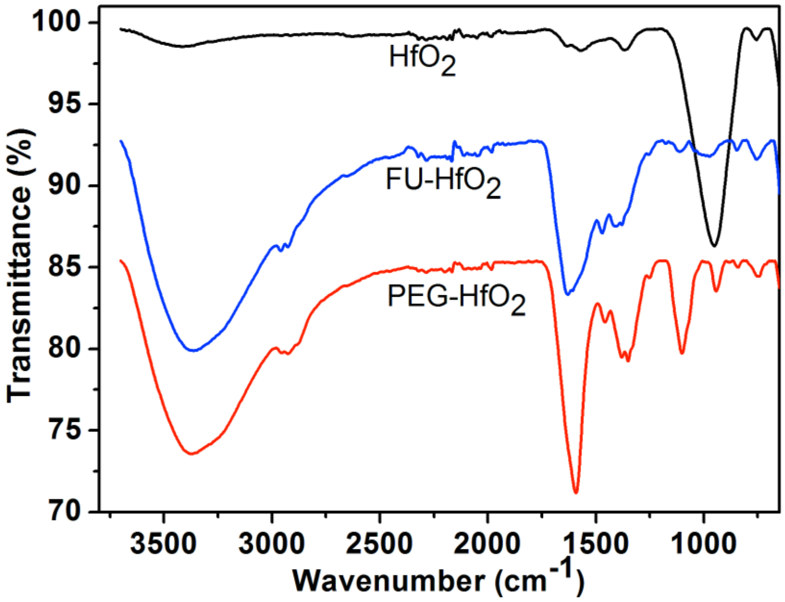

Figure S1. FTIR transmission spectra of cubic HfO_2 nanostructures.

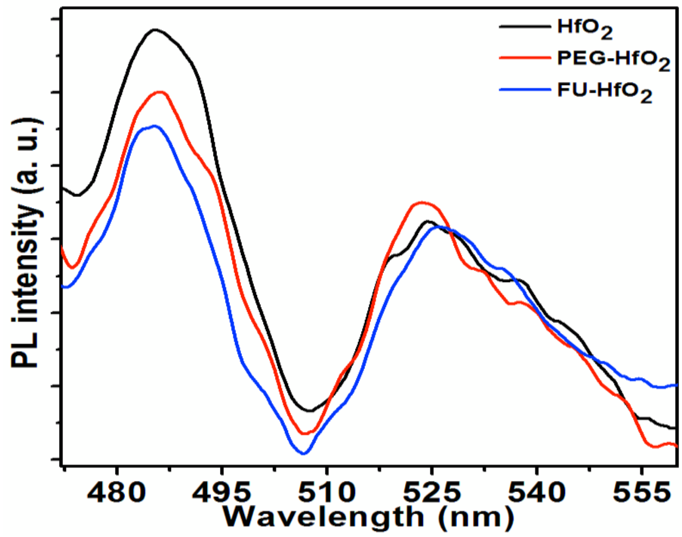

Figure S2. Photoluminescence (PL) spectra of cubic HfO_2 nanostructures recorded at room temperature.

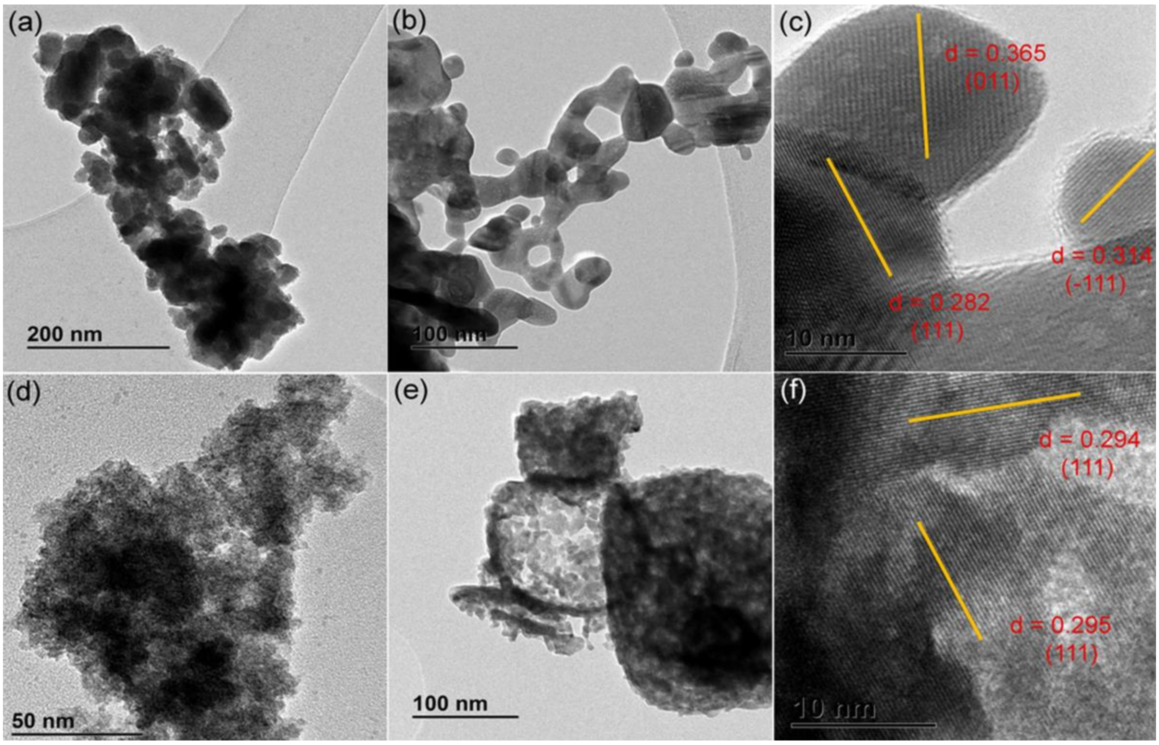

Figure S3. TEM micrographs of as-prepared HfO_2 (a) and after calcining at 550 °C (b, c) using NaOH (4 mmol); as-prepared HfO_2 (d) and after calcining at 550 °C (e, f) using a high concentration of NaOH (6 mmol).

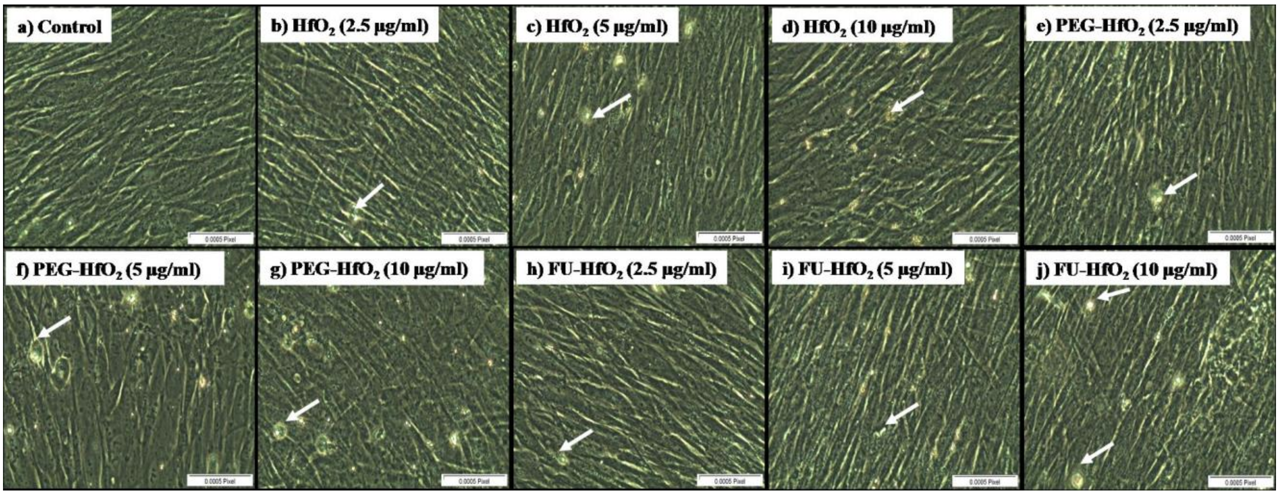

Figure S4. Morphological changes of WS1 cells after treatment with HfO_2, PEG-HfO_2, and FU-HfO_2 nanostructures; (arrows indicate the cell death).

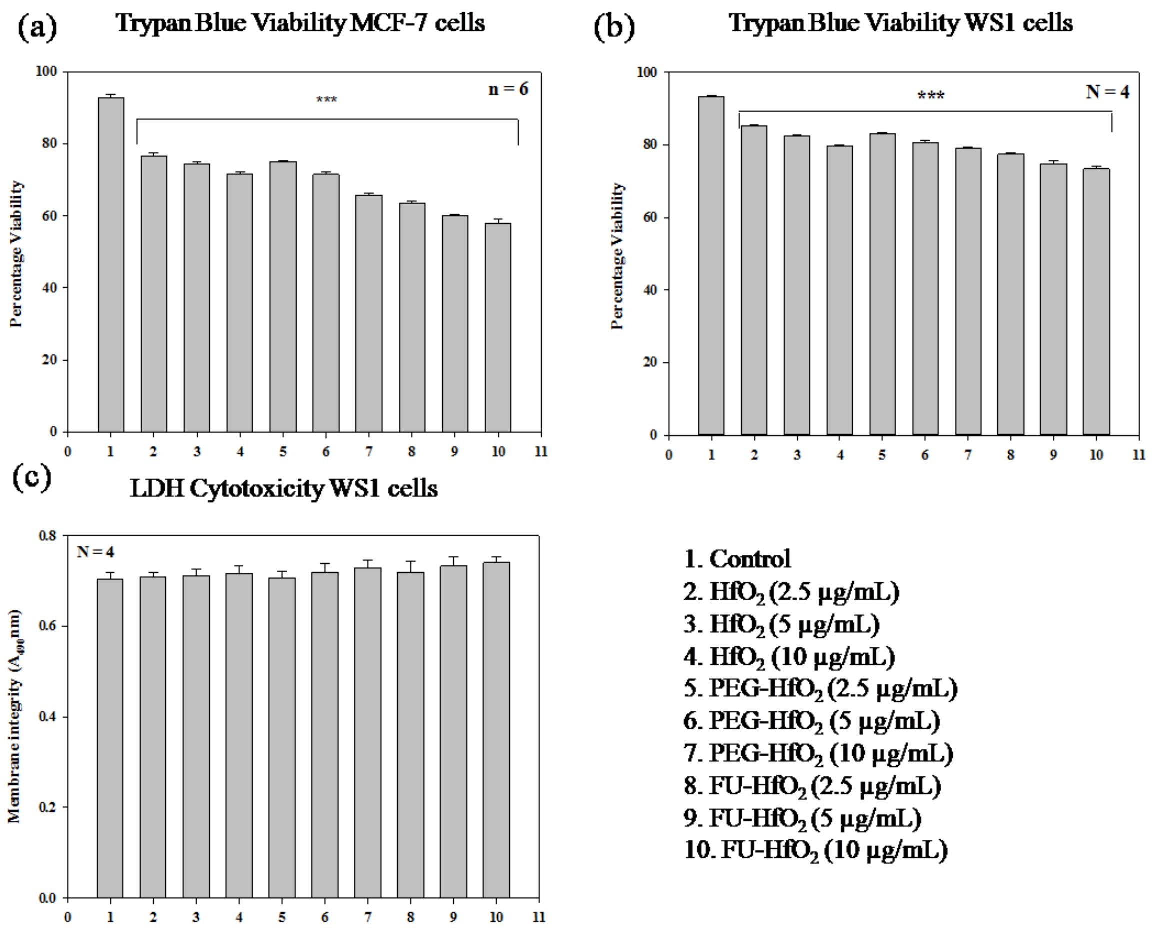

Figure S5. Effect of HfO_2, PEG-HfO_2, and FU-HfO_2 nanostructures on: (a) Trypan blue viability of MCF-7 cells, (b) Trypan blue viability of WS1 cells, and (c) LDH cytotoxicity of WS1 cells. Statistical significance values between the control and treated cells are shown as P <0.001 (***).

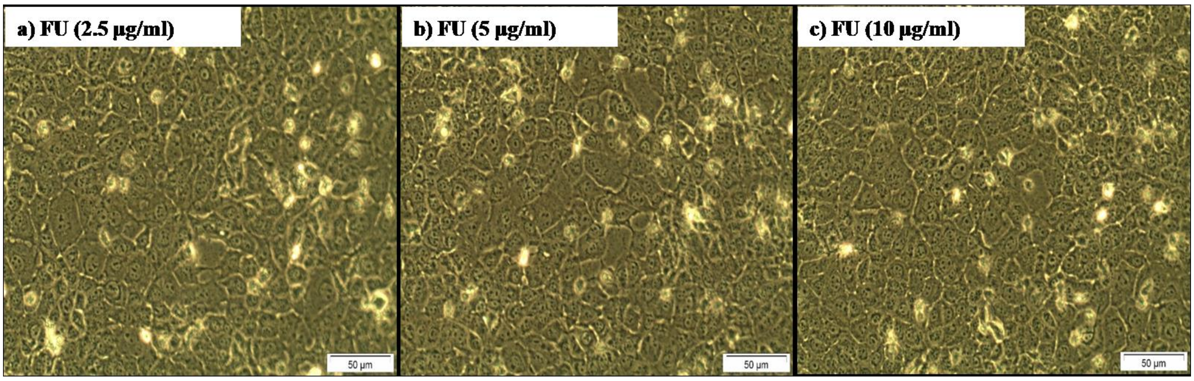

Figure S6. Morphological changes of MCF-7 cells after treatment with 5-fluorouracil (FU) (scale bar for all images is 50 µm).

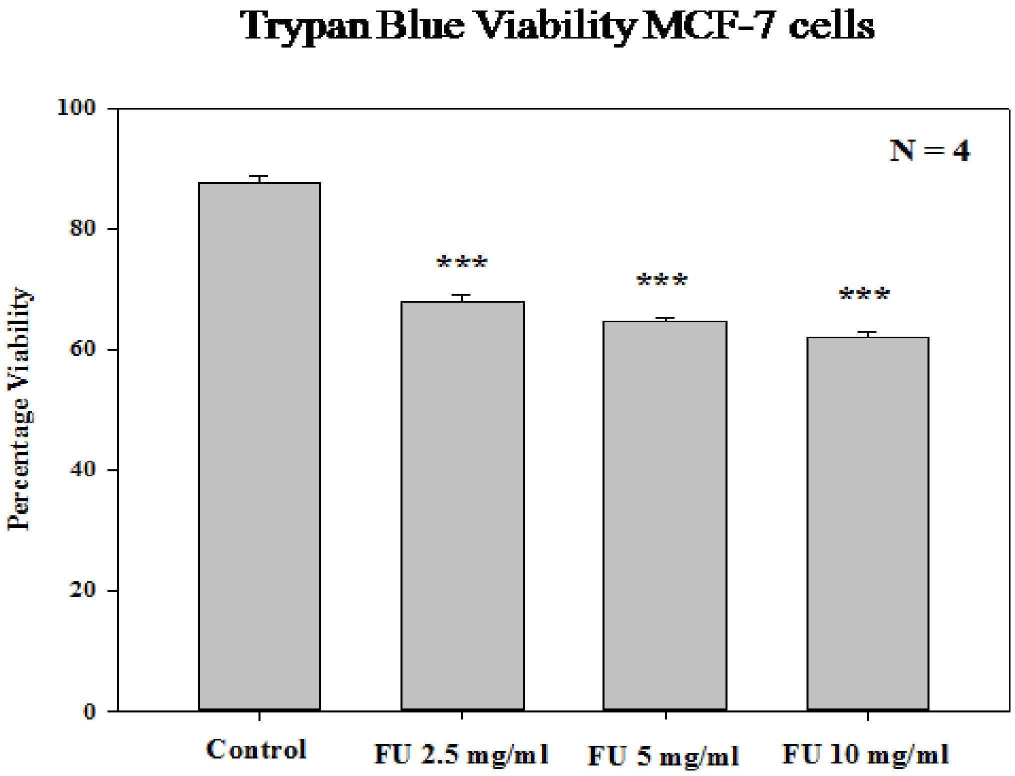

Figure S7. Effect of 5-fluorouracil (FU) on: (a) Trypan blue viability of MCF-7 cells. Statistical significance values between the control and treated cells are shown as P <0.001 (***).

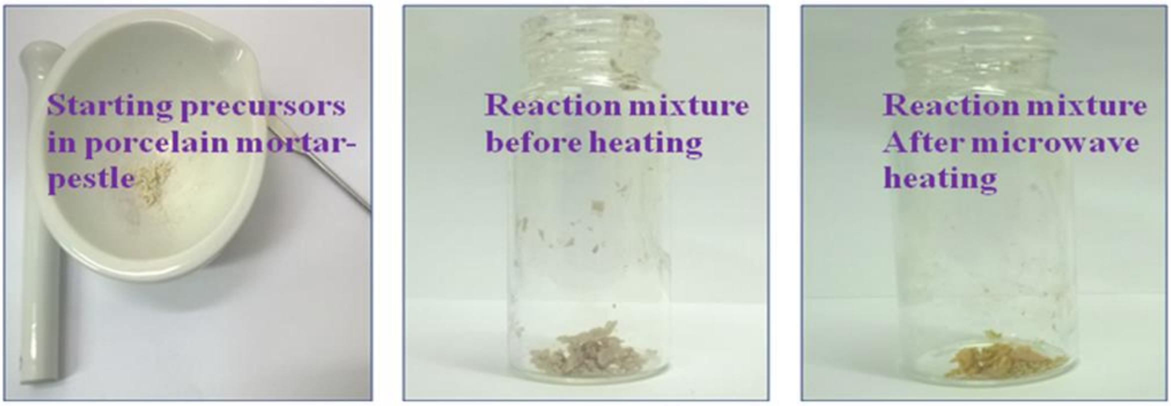

Figure S8. Digital images represent the different synthesis stages of cubic HfO_2 nanostructures.

Table S2. Summarised reaction conditions for the synthesis of cubic HfO_2 nanostructures

Hf Source	Reducing agent (mmol)	Calcination Temp. (°C)	Crystallinity	Product Formation	Morphologies
$HfCl_4$	**$Na_2S.xH_2O$**				
	0	-	Amorphous	Hafnium chlorate	-
	1	-	Amorphous	c-HfO_2	-
	3	-	Crystalline	c-HfO_2	Spherical and ellipsoidal shapes
	5	-	Crystalline	c-HfO_2/ m-HfO_2	-
	3 + D. I. water	-	Amorphous	c-HfO_2	-
	3, without washing	-	Crystalline	c-HfO_2+NaCl+ $Na_2S_2O_3$	-
	3	550 °C (1 h)	Crystalline	m-HfO_2/ c-HfO_2	-
	3	550 °C (3 h)	Crystalline	m-HfO_2	-
$HfCl_4$	**NaOH**				
	4	-	Amorphous	-	Aggregated nanoparticles
	4	550 °C (2 h)	Crystalline	m-HfO_2	Irregularly interconnected particles
	6	-	Amorphous	-	Aggregated nanoparticles
	6	550 °C (2 h)	Crystalline	c-HfO_2	Hollow spheres and clustered nanoparticles
	8	-	Amorphous	-	-
	8	550 °C (2 h)	Crystalline	c-HfO_2	-

m = monoclinic, c = cubic
